# Telemonitoramento da Insuficiência Cardíaca – A Experiência de um Centro

**DOI:** 10.36660/abc.20201264

**Published:** 2022-01-11

**Authors:** Isabel O. Cruz, Susana Costa, Rogério Teixeira, Fátima Franco, Lino Gonçalves

**Affiliations:** 1 Departamento de Medicina Interna Hospital Pedro Hispano Matosinhos Portugal Departamento de Medicina Interna , Hospital Pedro Hispano , Matosinhos - Portugal; 2 Departamento de Cardiologia Centro Hospitalar e Universitário de Coimbra Coimbra Portugal Departamento de Cardiologia , Centro Hospitalar e Universitário de Coimbra , Coimbra - Portugal

**Keywords:** Insuficiência Cardíaca/fisiopatologia, Telemonitoramento, Hospitalização, Serviços de Emergência

## Abstract

**Fundamento:**

A evolução natural da insuficiência cardíaca é uma pior progressiva e internações hospitalares recorrentes. São necessárias estratégias para se detectar descompensações em tempo hábil. O uso do telemonitoramento da insuficiência cardíaca é inconsistente.

**Objetivos:**

Este estudo tem o objetivo de avaliar o impacto desse programa de telemonitoramento (PTM) em internações hospitalares e admissões em serviços de emergência.

**Métodos:**

Este é um estudo retrospectivo observacional que analisou dados de todos os pacientes que se cadastraram no PTM de janeiro a 2018 a dezembro de 2019. Foram coletados dados demográficos, clínicos e relacionados ao PTM. O número de internações hospitalares e admissões em serviços de emergência do ano anterior e posterior ao cadastro foram comparados, utilizando-se o teste de Wilcoxon. Um p-valor bilateral de <0,05 foi considerado significativo.

**Resultados:**

Um total de 39 pacientes foram cadastrados, com uma média de idade de 62,1 ± 14 anos e predominância de pacientes do sexo masculino (90%). As causas mais comuns de insuficiência cardíaca foram cardiomiopatia isquêmica e dilatada. A fração de ejeção média foi de 30% e o tempo mediano da duração da doença foi de 84 meses (FIQ 33-144). Pacientes que foram cadastrados por menos de um mês foram excluídos, com um total de 34 pacientes analisados. Os pacientes foram acompanhados no PTM por um período mediano de 320 dias. O número de admissões em serviços de emergência foi reduzido em 66% (p<0,001) e o número de internações hospitalares por insuficiência cardíaca foi reduzido em 68% (p<0,001). O PTM não teve impacto no número de internações hospitalares por outras causas.

**Conclusões:**

Este estudo sugere que o PTM poderia reduzir a utilização de serviços de saúde em pacientes com insuficiência cardíaca.

## Introdução

A insuficiência cardíaca (IC) é um grande problema de saúde pública, cuja incidência tem aumentado, e com mortalidade e morbidade significativas. ^1^ Em Portugal, estima-se que a prevalência seja de 4,36%. ^2^ A evolução natural dessa doença é a piora dos sintomas e a diminuição da capacidade funcional com o tempo, com episódios de descompensação aguda, que geralmente levam à internação hospitalar. Após a primeira admissão, até 50% dos pacientes são readmitidos no período de seis meses após a alta ^3^ e entre 17% e 45% morrem durante o primeiro ano. ^4^ Internações hospitalares repetidas por IC têm um impacto negativo no prognóstico, sendo um preditor independente de mortalidade. ^5^

As principais estratégias para evitar a internação hospitalar são o tratamento farmacológico adequado, a educação do paciente, o acompanhamento estruturado e o automonitoramento. ^6^ Várias estratégias foram tentadas para se identificar os sinais de deterioração clínica, permitindo que houvesse uma intervenção antes da exacerbação aguda. O telemonitoramento é o uso da tecnologia para monitorar remotamente os pacientes em casa. ^7^ Ele foi proposto pela primeira vez nas Diretrizes da Sociedade Europeia de Cardiologia ( *European Society of Cardiology* – ESC) de 2016 para o diagnóstico e tratamento de IC aguda e crônica, com recomendação de classe II, com nível de evidência B. ^6^ Estudos demonstraram que programas de telemonitoramento poderiam reduzir os índices de internações hospitalares e admissões em serviços de emergência, durações dos períodos de internação, e até mesmo a mortalidade relacionada à IC. ^8 - 11^ Outras possíveis vantagens são o envolvimento dos pacientes e das famílias no controle da doença, a otimização do tratamento médico em tempo hábil, o aumento da adesão ao tratamento e a melhoria da qualidade de vida do paciente. ^9^ No entanto, esses resultados são inconsistentes, dado que outros estudos apresentam resultados nulos. ^12 , 13^ Isso provavelmente se deve às diferenças entre as populações estudadas, os sistemas de saúde, e os tipos de telemonitoramento. ^14^

## Objetivos

O objetivo deste estudo é avaliar o impacto do telemonitoramento remoto não invasivo em pacientes portugueses com insuficiência cardíaca avançada em internações hospitalares e admissões em serviços de emergência.

## Materiais e métodos

### Desenho do estudo

Este é um estudo retrospectivo observacional de antes e depois de pacientes cadastrados em um programa avançado de telemonitoramento (PTM) de insuficiência cardíaca. Isso significa que cada paciente tem seu próprio controle, comparando eventos no ano anterior à entrada no programa e durante o período de PTM.

### Seleção dos pacientes

Para serem incluídos no PTM, os pacientes precisavam ter mais de 18 anos, ter diagnóstico de IC e ser capazes de lidar com os dispositivos médicos. Selecionamos dados sobre pacientes que se cadastraram no programa entre 1º de janeiro de 2018 e 30 de novembro de 2019. Participantes acompanhados por menos de um mês foram excluídos da análise.

### Protocolo de telemonitoramento

O processo de telemonitoramento consistiu em medições diárias de dados fisiológicos, especificamente peso corporal, pressão arterial, frequência cardíaca, saturação de oxigênio e temperatura corporal, além da realização de um eletrocardiograma de três derivações semanalmente.

Após a seleção, os pacientes e cuidadores passaram por treinamento sobre como usar os dispositivos e transmitir os dados. Os valores basais foram definidos como uma mediana dos três primeiros valores registrados. Os desvios que disparariam um alerta foram definidos pela equipe médica ( Tabela 1 ).

**Tabela 1:** – Definição das variáveis relatadas e desvios que disparam um alerta

	Normal	Alerta
Saturação periférica de O _2_ (SpO _2_ )	∆ <4% da basal	∆ ≥4% e SpO _2_ <92%
Frequência Cardíaca	50-100 ppm	<50 ou >100
Pressão arterial sistólica	∆ ≤20% da basal	∆ >20% da basal
Pressão arterial diastólica	∆ ≤20% da basal	∆ >20% da basal
Peso corporal	∆ <1Kg	∆ ≥1 Kg em 24h ou ≥2Kg em 3 dias
Temperatura	≤37,5 ºC	>37,5 ºC
Eletrocardiograma de três derivações	FC 50-100	FC <50 ou >100

*FC: Frequência cardíaca. Kg: quilograma.*

Foram definidos dois tipos de alerta. Alertas técnicos aconteciam se houvesse falha na transmissão de dados ou se não houvesse relato de dados, e estes eram resolvidos por um técnico. Alertas clínicos foram uma medida acima ou abaixo dos limites pré-definidos. Um enfermeiro ligaria para os pacientes com alerta clínico para perguntar sobre os sintomas e aplicar o teste de Morisky-Green para medir a aderência aos medicamentos. Se o alerta clínico fosse validado, o médico responsável entraria em contato com o paciente para decidir sobre a melhor estratégia de controle.

### Coleta de dados

Dados demográficos dos pacientes e características da doença foram obtidos de prontuários médicos eletrônicos. Coletamos dados sobre internação hospitalar global e por insuficiência cardíaca, bem como admissões em serviços de emergência, do ano anterior à entrada no programa e ao período de cadastro.

### Análise estatística

Os dados de linha de base foram resumidos utilizando-se estatísticas descritivas: médias e desvio padrão para dados contínuos com distribuição normal, medianas e faixas interquartis para dados enviesados, e porcentagens para dados categóricos. O teste de Shapiro-Wilk foi usado para determinar a normalidade da distribuição. A análise comparativa entre o cadastramento antes e depois do programa foi feita utilizando-se o teste de Wilcoxon. Um p-valor bilateral de <0,05 foi considerado significativo. Todas as análises foram realizadas utilizando-se o software de análise de dados e estatística (SPSS).

## Resultados

Um total de 39 pacientes foram incluídos no programa. As características da linha de base são apresentadas na tabela 2 . A média de idade foi de 62 anos, variando entre 34 e 90 anos de idade. Houve uma predominância de pacientes do sexo masculino. A maioria dos pacientes tinha cardiomiopatia isquêmica ou dilatada, com uma duração mediana da doença de 84 meses (FIQ: 33-144). A fração de ejeção ventricular esquerda média foi de 29%± 9,3% e nenhum paciente apresentou fração de ejeção preservada. A fibrilação atrial estava presente em mais de dois terços dos pacientes.

**Tabela 2:** – Características de linha de base da população

n=34*	
Idade, anos	62 ± 14
**Sexo masculino, n (%)**	**30 (88%)**
Etiologia, n (%)	
Doença cardíaca isquêmica	13 (38,3%)
Cardiomiopatia dilatada	14 (41,2%)
Relacionada ao consumo de álcool	3 (8,9%)
Idiopática	7 (20,7%)
Pós-quimioterapia	1 (2,9%)
Pós-miocardite	2 (5,8%)
Familiar	1 (2,9%)
Cardiomiopatia hipertrófica	3 (8,9%)
Miocárdio não compactado de ventrículo esquerdo	1 (2,9%)
Doença cardíaca congênita	1 (2,9%)
Displasia arritmogênica do ventrículo direito	1 (2,9%)
Amiloidose	1 (2,9%)
Fração de ejeção ventricular esquerda, n (%)	
Normal (>50%)	0 (0%)
Disfunção leve (40-50%)	8 (23,5%)
Disfunção moderada (30-40%)	9 (26,5%)
Disfunção grave (<30%)	17 (50%)
Classe NYHA, n (%)	
I	0 (0%)
II	15 (44,1%)
III	18 (53%)
IV	1 (2,9%)
Fibrilação atrial, n (%)	22 (64,7%)
Medicamentos, n (%)	
Betabloqueador	30 (88,2%)
Inibidores da ECA ou BRA	12 (35,3%)
Inibidores da NRA	15 (44,1%)
Antagonista do receptor da aldosterona	31 (91,2%)

NYHA: New York Heart Association; ECA: enzima conversora da angiotensina; BRA: bloqueador do receptor de angiotensina; NRA: neprilisina receptor de angiotensina. * Dados apresentados como média ± desvio padrão ou n, (%)

Os pacientes foram acompanhados por um período mediano de 320 dias (FIQ: 166-486). Durante esse período, três pacientes abandonaram o estudo, um foi submetido a um transplante cardíaco, e cinco pacientes faleceram enquanto estavam no programa. As causas de morte foram a piora da IC em dois pacientes e infecção em três outros casos. Para esses sujeitos, todos os eventos que antecederam ao momento da interrupção foram levados em conta na análise principal. Os demais 25 sujeitos ainda estavam cadastrados no PTM no momento da análise.

A adesão foi boa, com 58% dos pacientes relatando todos os dados pelo menos 75% do tempo, e 75% dos pacientes relatando pelo menos um parâmetro mais de 75% do tempo.

**Figura 1:**
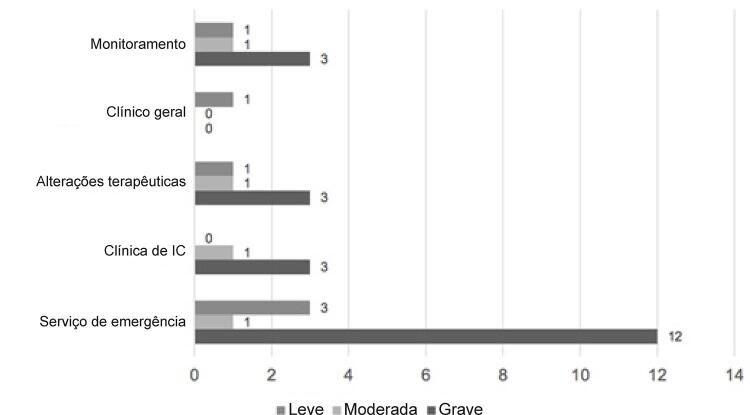
– Gerenciamento da decisão médica após alerta clínico relevante e contato telefônico com o paciente.

Houve um total de 2928 alertas clínicos, porém apenas 31 foram confirmados como sendo clinicamente relevantes, especialmente as mudanças no peso e na frequência cardíaca. Os 98,9% restantes dos alertas foram considerados não significativos devido à ausência de sintomas ou medições incorretas. Os alertas significativos são definidos como leves, moderados ou graves, de acordo com o desvio dos limites pré-definidos. Nesses casos, o médico ligaria para o paciente e decidiria sobre o controle, conforme ilustrado na figura 1 . Metade dos pacientes foram atendidos no departamento de emergência; entretanto, os demais casos foram resolvidos por alterações terapêuticas, uma consulta na clínica da IC, ou apenas o monitoramento.

**Figura 2:**
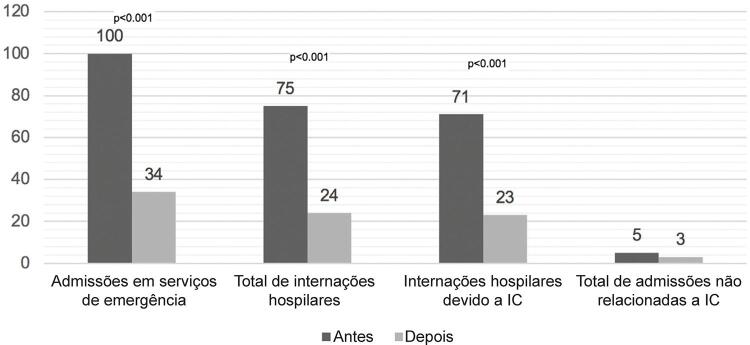
– A análise comparativa pelo teste de Wilcoxon revelou redução significativa nas admissões em pronto-socorro e nas internações por IC, ao comparar os números do ano anterior com os números durante a adesão ao programa de telemonitoramento.

O número total de consultas em serviços de emergência diminuiu de 100 admissões, no ano anterior ao cadastro, para 34, durante o programa de telemonitoramento (redução de 66%, p <0,001). As internações hospitalares por IC diminuíram de 71 para 23 (68%, p<0,001) e o número de dias no hospital também diminuiu significativamente, de 692 dias para 178 dias. Não foram encontradas diferenças nas admissões devido a outras causas. Esses resultados estão ilustrados na figura 2 .

Nenhum evento adverso foi causado pelo sistema de monitoramento.

## Discussão

Recentemente, o telemonitoramento domiciliar tem aparecido como uma opção adicional no controle de IC, disponibilizando sinais vitais e sintomas regulares e confiáveis de pacientes da comunidade. Este é o primeiro estudo a testar essa hipótese na população portuguesa. Encontrou-se uma redução significativa de 68% nas internações hospitalares devido à piora da IC e uma redução de 66% em consultas globais em serviços de emergência. Outro ponto importante é a redução do período de estadia no hospital, provavelmente resultante tanto da identificação precoce da descompensação e da alta antecipada, com o monitoramento próximo do paciente. Isso é particularmente relevante devido à limitação de leitos hospitalares. Esses resultados corroboram estudos anteriores que demonstraram os benefícios do telemonitoramento remoto.

O monitoramento diário do peso é uma recomendação de classe I no controle da IC ^6^ , já que a retenção de líquidos é um sinal de piora e não adesão ao tratamento diurético. Em nosso estudo, esse foi o alerta clínico mais comumente significativo identificado, levando a uma intervenção, principalmente alterações terapêuticas, consultas clínicas, ou admissões em serviços de emergência. Entretanto, Zhang et al., ^15^ demonstraram que a pesagem isoladamente tinha valor limitado.

Em 2019, os programas de telemonitoramento foram endossados ainda mais pela Sociedade Europeia de Cardiologia (ESC), ^16^ principalmente devido a duas publicações. O estudo TIM-HF2 ^17^ demonstrou que uma avaliação domiciliar regular de peso, pressão arterial, frequência cardíaca, saturação de oxigênio, eletrocardiograma e status geral da saúde poderia reduzir a proporção de dias de afastamento devido a internações hospitalares por problemas cardiovasculares (especialmente IC) não planejadas ou mortes (p = 0,046), bem como mortalidade global (FC 0,70; p = 0,028). Uma análise de Cochrane ^18^ de 25 estudos concluiu que o telemonitoramento reduziu a mortalidade global em 20%, e a internação hospitalar por IC em 30%.

A ESC afirmou que o protocolo usado no estudo TIM-HF2 deveria ser tentado em outros países, para testar sua capacidade de ser reproduzido. ^16^ A população participante do estudo TIM-HF2 foi similar à nossa em termos de distribuição por sexo, classe da NYHA, e etiologia da insuficiência cardíaca. Entretanto, sua população era 8 anos mais velha, e 25% de seus pacientes apresentavam fração de ejeção preservada, enquanto, em nosso estudo, não havia nenhum. O programa de telemonitoramento é muito semelhante ao nosso e nossos resultados positivos podem indicar que ele seja um método efetivo de monitoramento remoto.

O controle do paciente à distância não deve se limitar ao monitoramento dos sinais vitais. A equipe médica pode usar esses dados para individualizar o tratamento, oferecer educação ao paciente, e introduzir ou aumentar terapias que modificam a doença. Essa abordagem pode levar a um impacto mais significativo no prognóstico.

Em outros estudos, o grau de adesão variou de 80% a 90%, ^7 , 14^ que é um valor melhor que nossos números. Isso se deve provavelmente aos fatos de que os pacientes apresentaram menos adesão na prática clínica diária se comparado aos momentos de estudos clínicos. Em Portugal, conforme mostrado pelo HLS-EU-PT, ^18^ 61% da população pesquisada têm um nível de conhecimento de saúde geral inadequado. Isso pode ser uma barreira para um tratamento eficaz da doença, já que esses pacientes têm mais dificuldade de entender a doença e seu controle. ^19^ Alguns estudos também demonstraram que isso leva a uma baixa adesão aos medicamentos e ao aumento das internações hospitalares. Ao entrar nesse tipo de programa, os pacientes e cuidadores recebem mais conhecimento e responsabilidade sobre o controle da doença. ^20^ Isso provavelmente ajuda a explicar por que, embora o número de alertas clínicos significativos seja baixo, o número de internações hospitalares por IC diminuem grandemente.

Alguns estudos demonstraram que a idade não tem impacto nesses resultados, com pacientes acima de 75 anos de idade tendo o mesmo benefício que os pacientes mais jovens. ^21^ Isso é importante uma vez que temos uma população em envelhecimento, e com alta prevalência de IC e internações hospitalares frequentes.

As principais limitações deste estudo são aquelas associadas ao estudo antes e depois, principalmente a ameaça da evolução, que é definida como outros eventos que poderiam afetar os resultados. Nesse tipo de estudo, outras variáveis, como as alterações nos medicamentos ou outras intervenções, não são registradas. Também temos uma amostra pequena. Entretanto, este estudo ainda pode gerar evidência preliminar para a eficácia da intervenção em uma população com insuficiência cardíaca relativamente grave. Outra limitação é que as internações hospitalares que ocorrem em hospitais fora do Serviço Nacional de Saúde não foram registradas, embora não sejam incomuns.

Pesquisas posteriores devem se concentrar na identificação dos parâmetros biológicos mais importantes a serem monitorados, na definição dos subgrupos de pacientes que serão mais beneficiados por essa abordagem, e de quais são os programas com melhor relação custo-benefício.

## Conclusões

Nosso programa de telemonitoramento não invasivo reduziu as internações hospitalares e as admissões em serviços de emergência devido a IC, bem como o número de dias de internação por IC. A implementação de um programa desse tipo deve ser considerada para a melhoria dos resultados para pacientes com insuficiência cardíaca.
