# Supplementary material for: Telemonitoramento da Insuficiência Cardíaca – A Experiência de um Centro
Source: Arq Bras Cardiol. 2022 Jan 11;118(3):599–604. [Article in Portuguese] doi: 10.36660/abc.20201264 (PMC8959025; doi:10.36660/abc.20201264)
Supplement: Translation

# Translation: Telemonitoring in Heart Failure – A Single Center Experience

**Authors:** Isabel O. Cruz, Susana Costa, Rogério Teixeira, Fátima Franco, Lino Gonçalves

**Affiliations:** 1 Portugal Departamento de Medicina Interna, Hospital Pedro Hispano, Matosinhos - Portugal; 2 Portugal Departamento de Cardiologia, Centro Hospitalar e Universitário de Coimbra, Coimbra - Portugal

**Keywords:** Heart Failure/physiopathology, Telemonitoring, Hospitalization, Emergency Services

## Abstract

**Background:**

The natural history of heart failure is a progressive decline and recurrent hospital admissions. New strategies to timely detect decompensations are needed. The use of telemonitoring in heart failure is inconsistent.

**Objectives:**

This study aimed to evaluate the impact of this telemonitoring program (TMP) in hospitalizations and emergency department admissions.

**Methods:**

This is a retrospective observational study, that analyzed data of all the patients who enrolled in the TMP program from January 2018 to December 2019. Demographic, clinical, and TMP-related data were collected. The number of hospitalizations and emergency department admissions from the year before and after enrollment were compared, using the Wilcoxon test. A two-sided p<0.05 was considered significant.

**Results:**

A total of 39 patients were enrolled, with a mean age of 62.1 ± 14 years and a male predominance (90%). The most common causes of heart failure were ischemic and dilated cardiomyopathy. The mean ejection fraction was 30% and the median time of disease duration was 84 months (IQR 33-144). Patients who were enrolled for less than one month were excluded, with a total of 34 patients analyzed. Patients were followed in the TMP for a median of 320 days. The number of emergency department admissions was reduced by 66% (p<0.001), and the number of hospitalizations for heart failure was reduced by 68% (p<0.001). The TMP had no impact on the number of hospitalizations for other causes.

**Conclusions:**

This trial suggests that a TMP could reduce health service use in patients with heart failure.

## Introduction

Heart Failure (HF) is a major public health problem, with rising incidenc and a significant mortality and morbidity.^1^In Portugal, the prevalence is estimated to be 4.36%.^2^The natural history of this disease is worsening symptoms and diminishing functional capacity over time, with episodes of acute decompensation, often leading to hospital admissions. After the first admission, up to 50% of the patients will be readmitted within six months after discharge^3^and 17-45% will die in the first year.^4^Repeated hospitalizations for HF have a negative impact on prognosis, being an independent predictor of mortality.^5^

The main strategies to prevent hospitalization are adequate pharmacological treatment, patient education, structured follow-up, and self-monitoring.^6^Various strategies have been tried to timely identify signs of clinical deterioration, allowing for an intervention before the acute exacerbation takes place. Telemonitoring is the use of technology to remotely monitor patients at home.^7^It was first proposed in the 2016 European Society of Cardiology Guidelines for the diagnosis and treatment of acute and chronic HF, with a class II, level of evidence B recomendation.^6^Studies have shown that programs of telemonitoring could reduce the rate of emergency and hospital admissions, lengths of hospital stays, and even HF-related mortality.^8 - 11^Other possible advantages are involving patients and families in disease management, timely optimization of medical therapy, increased compliance, and improvement in the patients’ quality of life.^9^However, these results are inconsistent, as other studies show null results.^12 , 13^This is most likely due to differences in the study populations, healthcare systems, and types of telemonitoring.^14^

## Objectives

The aim of this study is to evaluate the impact of non-invasive remote telemonitoring in Portuguese patients with advanced heart failure in emergency and hospital admissions.

## Material and methods

### Study Design

This is a retrospective, observational, before and after study of patients enrolled in an advanced heart failure telemonitoring program (TMP). This means that each patient is his own control, comparing events in the one year before entering the program and during the period of TMP.

### Patient Selection

To be included in the TMP, patients had to be over 18 years of age, have a diagnosis of HF and be able to comply with the medical devices. We selected data on patients who enrolled in the program between January 1, 2018, and November 30, 2019. Participants followed up for less than a month were excluded from the analysis.

### Telemonitoring Protocol

The telemonitoring process consisted of daily measurements of physiological data, namely body weight, blood pressure, heart rate, oxygen saturation, and body temperature, plus a weekly performance of a three-lead electrocardiogram.

After selection, patients and caregivers received training on how to use the devices and transmit data. Basal values were defined as a median of the first three values registered. The deviations that would trigger an alert were defined by the medical team ( Table 1 ).

**Table 1:** – Definition of the variables reported and deviations that trigger an alert

	Normal	Alert
Peripheral saturation of O_2_(SpO_2_)	∆ < 4% of basal	∆ ≥ 4% and SpO_2_< 92%
Heart Rate	50-100 ppm	<50 or > 100
Systolic blood pressure	∆ ≤ 20% of basal	∆ > 20% of basal
Diastolic blood pressure	∆ ≤ 20% of basal	∆ > 20% of basal
Body weight	∆ <1Kg	∆ ≥ 1 Kg in 24h or ≥ 2Kg in 3 days
Temperature	≤ 37.5ºC	>37.5ºC
Three-lead electrocardiogram	HR 50-100	HR <50 or > 100

*HR: Heart rate. Kg: kilogram.*

Two types of alerts were defined. Technical alerts were failure on data transmission or no data reported, and they were fixed by a technician. Clinical alerts were a measurement above or below the preset limits. A nurse would call the patients with a clinical alert to ask about symptoms and apply the Morisky-Green test to measure medication adherence. If the clinical alert was validated, the referring physician would contact the patient to decide on the best management strategy.

### Data Collection

Data on patient demographics and disease characteristics were obtained from electronic medical records. We collected data on all-cause hospitalization and heart failure hospitalization, as well as emergency department admissions, from the year before entering the program and enrollment period.

### Statistical analysis

Baseline data were summarized using descriptive statistics: means and standard deviation for continuous data with normal distribution, medians and interquatile range for skewed data, and percentages for categorical data. The Shapiro-Wilk test was used to assess the normality of distribution. Comparative analysis between before and after enrollment in the program was done using the Wilcoxon test. A two-sided *p* <0.05 was considered significant. All analyses were performed using data analysis and statistical software (SPSS).

## Results

A total of 39 patients were included in the program. Baseline characteristics are presented in table 2 . The mean age was 62 years, ranging from 34 to 90 years old. There was a male predominance. Most patients had ischemic or dilated cardiomyopathy, with a median disease duration of 84 months (IQR: 33-144). Mean left ventricular ejection fraction was 29%± 9.3% and no patient presented with preserved ejection fraction. Atrial fibrillation was present in more than two-thirds of the patients.

**Table 2:** – Population baseline characteristics

n=34*	
Age, years	62 ± 14
**Male gender, n (%)**	**30 (88%)**
Etiology, n (%)	
Ischemic heart disease	13 (38,3%)
Dilated cardiomyopathy	14 (41,2%)
Alcohol-related	3 (8,9%)
Idiopathic	7 (20,7%)
Post-chemotherapy	1 (2,9%)
Post-myocarditis	2 (5,8%)
Familial	1 (2,9%)
Hypertrophic cardiomyopathy	3 (8,9%)
Left ventricular non-compaction cardiomyopathy	1 (2,9%)
Congenital heart disease	1 (2,9%)
Right ventricular arrhythmogenic dysplasia	1 (2,9%)
Amyloidosis	1 (2,9%)
Left ventricular ejection fraction, n (%)	
Normal (>50%)	0 (0%)
Mildly impaired (40-50%)	8 (23,5%)
Moderately impaired (30-40%)	9 (26,5%)
Severely impaired (<30%)	17 (50%)
NYHA class, n (%)	
I	0 (0%)
II	15 (44,1%)
III	18 (53%)
IV	1 (2,9%)
Atrial Fibrillation, n (%)	22 (64,7%)
Medications, n (%)	
Beta blocker	30 (88,2%)
ACE inhibitor or ARB	12 (35,3%)
ARN inhibitor	15 (44,1%)
Aldosterone-receptor antagonist	31 (91,2%)

NYHA: New York Heart Association; ACE: angiotensin- converting enzyme; ARB: angiotensin-receptor blocker; ARN: angiotensin receptor-neprilysin. * Data presented as mean ± standard deviation or n, (%)

Patients were monitored for a median of 320 days (IQR: 166-486). During that period, three patients dropped out of the program, one was submitted to a heart transplant, and five died while on the program. The causes of death were worsening HF in two patients and infection in the other three. For these subjects, all events up to the time of discontinuation were accounted for in the main analysis. The remaining 25 were still enrolled in the TMP by the time of this analysis.

Compliance was good, with 58% of patients reporting all data at least 75% of the time and 75% reporting at least one parameter for more than 75% of the time.

There were a total of 2,928 clinical alerts, but only 31 were confirmed as clinically relevant, mainly changes in weight and heart rate. The remaining 98.9% alerts were considered non-significant due to the absence of symptoms or wrong measurements. The significant alerts are defined as mild, moderate, or severe, according to the deviation from the preset limits. In these cases, the doctor would call the patient and decide on management, as represented in figure 1 . Half of the patients were observed in the emergency department, but the remaining cases were resolved by therapeutic changes, a visit to the HF clinic, or simple monitoring.

**Figure 1:** – Medical decision management after a relevant clinical alert and telephone contact with the patient.

The total number of emergency department visits decreased from 100 admissions in the year prior to the enrollment, to 34 during the telemonitoring program (reduction of 66%, p <0.001). Hospitalizations for HF decreased from 71 to 23 (68%, p<0.001) and the number of days in hospital also decreased significantly, from 692 days to 178 days. No differences were found in admissions due to other causes. These results are represented in figure 2 .

**Figure 2:** – Comparative analysis using the Wilcoxon test revealed a significant reduction in emergency room admissions and hospitalizations due to heart failure, when compare the numbers from the previous year with the numbers when joining the telemonitoring program.

No adverse events were caused by the monitoring system.

## Discussion

Recently, home-based telemonitoring has emerged as an add-on option to HF management, providing regular and reliable vital signs and symptoms of community-based patients. This is the first study to test this hypothesis in the Portuguese population. We found a 68% significant reduction in hospitalizations due to worsening HF and a 66% reduction in emergency department visits for all-causes. Another important point is the reduction in hospital length of stay, probably the result of both the early identification of the decompensation and early discharge with close monitoring of the patient. This is particularly relevant due to the limitation of beds in the hospitals. These results support previous studies that have shown benefits from remote telemonitoring.

Daily weight monitoring is a class I recommendation in the management of HF,^6^because fluid retention is a sign of clinical decline and non-compliance to diuretic treatment. In our study, this was the most commonly significant clinical alert identified, leading to an intervention, mainly therapeutic changes, clinical visits, or emergency department admissions. However, Zhang et al.^15^demonstrated that measuring weight alone has limited value.

In 2019, telemonitoring programs were further endorsed by the European Society of Cardiology (ESC),^16^mainly due to two publications. The TIM-HF2 trial^17^demonstrated that a regular home assessment of weight, blood pressure, heart rate, oxygen saturation, electrocardiogram, and general health status could reduce the proportion of days lost due to unplanned CV (mainly HF) hospitalizations or death (p = 0.046) as well as all-cause mortality (HR 0.70; p = 0.028). A Cochrane review^18^of 25 trials concluded that telemonitoring reduced all-cause mortality by 20% and HF hospitalization by 30%.

The ESC stated that the protocol used in the TIM-HF2 trial should be tried in other countries to test for reproducibility.^16^The population studied in TIM-HF2 trial was similar to ours in terms of gender distribution, NYHA class and heart failure etiology. However, their population was 8 years older and they had 25% of patients with preserved ejection fraction, while we had none. The telemonitoring program is very similar to our own and our positive results may indicate that it is an effective method of remote monitoring.

Distant patient management should not be limited to monitoring vital signs. The medical team can use that data to individualize care, provide patient education, and timely introduce or uptitrate disease-modifying therapies. This approach might lead to a more significant impact on prognosis.

In other studies, the degree of compliance ranged from 80% to 90%,^7 , 14^which is better than our numbers. This is probably because patients are less compliant in everyday clinical practice than in clinical trials. In Portugal, as shown by the HLS-EU-PT,^18^61% of the surveyed population has an inadequate general health literacy level. This can be a barrier to an effective disease treatment, as these patients have a more difficulty understanding of the disease and its management.^19^Some studies have also shown that it leads to poor medication adherence and increased hospitalization. By entering such a program, patients and caregivers, are given more knowledge and responsibility on disease management.^20^This can probably help to explain why, even though the number of significant clinical alerts is low, the number of hospital admissions for HF reduces greatly.

Some studies have shown that age does not have an impact on these results, with patients over 75 years of age having the same benefit as younger patients.^21^This is important because we have an aging population with a high prevalence of HF and frequent hospital admissions.

The main limitations of this paper are those associated with a before-and-after study, mainly history threat, that is defined as other events that could affect outcomes. In this type of study other variables, such as medication changes or other interventions, are not recorded. We also have a small sample size. However, this study can still produce preliminary evidence for intervention effectiveness in a population with relatively severe heart failure. Another limitation is that admissions occurring in hospitals outside the National Health System were not recorded, although they are uncommon.

Further research should focus on identifying the most important biological parameters to monitor, which subgroups of patients will benefit the most from this approach and which are the most cost-effective programs.

## Conclusions

Our non-invasive telemonitoring program has significantly reduced HF hospitalizations and emergency department admissions, as well as days in hospital for HF. Implementation of such a program should be considered to improve the outcomes for patients with heart failure.
